# Hospital Meetings

**Published:** 1902-03-01

**Authors:** 


### HOSPITAL MEETINGS.

### CITY OF LONDON LYING-IN HOSPITAL.

THE 151st annual meeting of the City of London Lying-in Hospital was held on Wednesday, 19th inst., the Rev. G. H. PERRY presiding. As usual the attendance was very limited, those present consisting chiefly, if not entirely, of members of the committee and staff.

The report presented by the Committee of Management stated that the year 1901 had been a satisfactory one both as regarded the sanitary condition of the hospital and its financial position. During the year 650 women were delivered in the hospital, being 51 more than in 1900, and the highest number ever admitted in one year. There were only two maternal deaths, the first being on March 4th, from embolism, and the other on November 26th, 21 days after delivery, from septicæmia accelerated by bronchitis and heart disease. The 650 women gave birth to 655 children, viz., 333 boys and 322 girls, 5 women had twins, 31 children were stillborn, 9 were apparently stillborn, but restored, and 15 died. The daily average number of beds occupied was 27·5, and the average stay of each patient 15·5 days, as against 26·5 and 16 respectively in 1900. The average daily number of inmates was 75, as against 72·8 last year. In the out-patient department there was a very considerable increase, 2,051 women being delivered at their homes, as against 1,753 in 1900, and 1,559 in 1899; 2,080 children were born: of these 1,085 were boys and 995 girls, 27 women had twins, and one woman was delivered of triplets, 72 children were stillborn, and 17 apparently so, though subsequently restored, 3 women and 35 children died. The midwife pupils, with the district surgeons and resident outdoor midwife, under whose supervision they worked, attended 825 of the above cases without a single death. There were 48 midwives and 144 monthly nurses trained in the hospital during the past year; 45 of the midwives presented themselves for the London Obstetrical Society's examination, and with three exceptions passed, and received certificates of competence. The new rules for pupils training in the hospital, which came into force at the commencement of the year, worked satisfactorily and proved advantageous. The applications were so numerous that pupils had to enter their names four or five months before they could be received. The income for the year was £4,893 and the expenditure £3,358, the endowment fund having been increased by the purchase for £1,491 of £1,500 India 3 per Cents. The capital of the Samaritan Fund had also been increased by the purchase of £250 Consols and now amounted to £1,750.

THE BUILDING UNDERMINED BY TUBE RAILWAYS.

The committee expressed regret that since the commencement of the works of the Great Northern and City Railway Company and the City and South London Railway Company, and owing to the character thereof, a considerable portion of the hospital buildings had suffered very seriously, and some portions were now in a condition necessitating extraneous support in order to make them temporarily as secure as the circumstances would permit. The committee, however, were advised that it would soon become imperatively necessary to incur very considerable expenditure for the purpose of restoring the buildings to the condition in which they were prior to the operations of the railway companies, and they were taking advice as to their legal rights against those companies. Since the last annual meeting Mr. H. Clifford Gosnell and Mr. C. G. B. Marsham had joined the Committee of Management Dr. H. Meggett, district surgeon for South Tottenham, having resigned his appointment, in consequence of leaving the neighbourhood, Dr. Sheperd had been appointed to succeed him.

The CHAIRMAN, in moving the adoption of the report drew attention to the many satisfactory features it contained. Mr. W. P. RUSSELL seconded, and said the only subject of anxiety was the state of the building; but they hoped in the result to get it restored without a large draft on their funds. The report was adopted, as was also that of the medical staff, with a vote of thanks for their services.

The Committee of Management was re-elected, it being left to the committee to fill up the vacancies caused by the death of Mr. Lloyd, and the resignation of Mr. Wilson, who had been appointed solicitor. The Finance Committee, the honorary officers, and Dr. Yarrow, the surgeon-accoucheur, were re-elected, and the customary vote of thanks to the chairman, closed the proceedings.

### QUEEN CHARLOTTE'S HOSPITAL.

AN UNEVENTFUL YEAR.

THAT nothing of unusual interest was expected to happen at the annual meeting on Monday afternoon was evident, for the customary practice of preparing a draft report of the meeting was adhered to on this occasion. The only alteration made, before the report was signed by the chairman, was that a resolution, thanking Her Majesty Queen Alexandra for her consent to become patroness in succession to the late Queen Victoria, was placed first in the list. The Queen has also become an annual subscriber. Nine governors were present, with the Earl of Hardwicke as chairman; they adopted the annual report, passed votes of thanks to the honorary officers, and elected some fresh governors. Neither is the annual report an eventful one, and only two matters call for special remark. The first relates to finance, and is this: that the committee have been obliged to use the legacies, amounting to £1,574 12s., for expenses incurred in the enlargement of the hospital, instead of investing them. This has been the case for the past five years, and it is therefore not surprising that Lord Hardwicke should have called attention, in his remarks on the report, to the need for additional annual subscribers. The total sum received for the Enlargement Fund, including the transferred legacies, is £5,507 18s. 1d., which is "still far short of the expense that has been incurred."

The second point is that, after conferring with representatives of other Lying-in Hospitals in London, the committee have taken steps to increase the period of training for midwives and monthly nurses to five months. This has not been made compulsory, but as a result of the new arrangement. A large number of pupils have already offered themselves for the longer course.

### ST. MARY'S HOSPITAL, PLAISTOW: THE TURNING POINT.

The governors of the little hospital for sick children, formerly known as St. Mary's Day Nursery, Plaistow, evidently take a keen interest in its progress. Eleven attendances at the annual meeting is not at all a bad proportion for such an out-of-the-way neigbourhood; and one is inclined to rate devotion to local improvements more highly in a new and—it must be confessed—somewhat dreary-looking neighbourhood than in one that bears the stamp of well-established prosperity on its face. Several of the governors, fearing there might not be a quorum, had made a special effort to be there on Thursday afternoon, and among these was the Bishop of Barking, chairman, whose duties are many, and who had several other meetings to attend that day.

### Royal Patronage.

The business before the meeting was the annual report, which opens with the statement that Her Most Gracious Majesty Queen Alexandra has, during the past year, signified her sympathy in the work of the institution by consenting to occupy the position of patroness. This, the committee of management feel, must be the turning point in the history of the hospital. Another point worthy of note is that to obviate the lack of cubic air-space in the wards, to which in sending their grant the hon. secretaries of the Prince of Wales's Fund drew attention in 1900, the number of beds has been reduced from 38 to 35. There has been another change of matron—Miss T. A. Bodington, who was appointed a year ago, has left, and her place has been taken by Miss Beatrice A. Cookes, formerly assistant matron at Preston Infirmary.

### The Event of the Day.

Just before the meeting began the secretary, Mr. Percy Glenton, who is quite as keen as any of the governors to see the little hospital grow, was deploring the small sum—only £144 14s. 9d.—received for the rebuilding since the last annual report was issued. Before the meeting ended, however, things had taken a turn for the better, for the Rev. T. Given Wilson, vicar of Plaistow, and moving spirit of the scheme, generously gave the site for erecting an entirely new hospital. This is close to the present hospital, and is now occupied by a mission hall. Thus, though the sum to be raised was after some discussion placed at double the original figure, a definite step towards a permanent building was made. It all came about when the last sentence in the report was read. There were, it was said, four great needs, the third being "Donations for the rebuilding of the hospital (about £12,000 will be needed for this purpose)." Mr. Given Wilson asked for a modification of that clause. He said that for the last month he had been taking advice from many friends, especially medical men (and Mr. Wilson himself had a medical training in his early days) as to the desirability of erecting, on the site adjoining the present building, a hospital for women and children. There seemed to be a concensus of opinion, that it was a magnificent site, and he thought £34,000 should be appealed for rather than £12,000. He purposed placing it under the trusteeship of the Bishop of St. Albans Trustees—it was now under the Charity Commissioners—for a hospital for women and children. It might perhaps be possible to get in a clause for a man's ward also. "Let the new building," said Mr. Given Wilson, "be quite up to date, and don't let us be too hasty. We shall get the money in time—nothing is more wanted than a hospital here—and then we can approach the Charity Commissioners about it. We can easily connect the two buildings, and then you have a scheme which will be one of the best things possible for the borough. I will give the site, and if I live I will do more."

One of the Governors thought the hospital should be for women and children only; they wanted more looking after than the men; and another asked whether it would be hurtful to the other hospitals.

Mr. Given Wilson replied that sometimes cases came to St. Mary's with which the medical men would be glad to deal; he did not suggest more than, say, four beds. And then, in reply to his question as to whether the doctors would not like it, they said they would.

The Chairman said he thought the meeting was unanimous in believing that it was a desirable thing to adopt the scheme, and that the clause at the end of the report should be recast. Some further discussion followed, on whether the site should be described as "given," "provided," or "promised" "We are justified," said the Chairman, addressing Mr. Given Wilson, "in thanking you for it?" "Oh, I don't want any thanks," was the reply; and finally the word "provided" was duly moved, seconded, and carried, as the word that best conveyed the idea of something tangible; and with one or two verbal improvements, the report for 1901 was adopted.

Several names "all good men and true," as the Chairman said, were proposed as members of the Management Committee, and the usual votes of thanks closed the meeting. A committee meeting was held immediately afterwards.

---

### TOTTENHAM HOSPITAL: A POINT OF ORDER.

There was a large attendance at the annual meeting of Tottenham Hospital on Tuesday afternoon, 24 governors being present, including one lady. A good deal of time was taken up in a discussion as to the sending out of the report, which had not been seen by the governors until they entered the room, when a limited number of copies were handed round. After the Chairman (the Hon. C. F. Cory-Wright) had run over the main points of the report, which it was decided to take as read, Mr. Joshua Adley said that he strongly objected to passing a report which he had not had time to look through. It was most inconvenient to have it handed to you when you got into the room. The whole thing was thus in the hands of the committee, and the subscribers and donors knew nothing about it. Last year the report was very incomplete, and he thought it was understood then that another year copies should be sent out before the meeting. It meant adopting a report they knew nothing about, but he supposed the committee thought that if they adopted it the meeting would too. Another governor said it was most absurd to hand round the report at twenty minutes past five, and expect it to be passed at once.

The Chairman said it was difficult to circulate the report very early, because it meant a great deal of expense—some £20—if it were not adopted. At this point there were sounds of disagreement from the governors. The Chairman, continuing, said the meeting was fixed so early in the year that it was very difficult to get the report ready in time—the auditors, for example, had only just completed their work. Still, he thought it might, perhaps, have been ready a week earlier. Last year he drew up the report himself; this year the committee did it, and that always meant delay. He would be only too glad if he could do anything to remedy it another year; but this year he must ask the meeting to accept it, because it was printed. If they wished, the clerk should read through the 20 pages. "No, no," the governors agreed.

Mr. Adley further objected that the names of the retiring members of committee did not appear in the report as eligible for re-election. This was done in every hospital and company he knew of. The Chairman replied that he believed he had seen the report of nearly every hospital in London, and it was not done.

Mr. Adley said: "It is a matter of common knowledge in all companies that certain directors retire, and the public ought to know who they are." The Chairman then acknowledged that the clerk ought to have placed the names on the agenda, or in the notice convening the meeting. If the meeting wished, he would insert a slip of paper stating that the retiring members were re-elected without opposition; this should be sent out with the report. A governor said: "And in future you will publish the names of the retiring members?" Another remarked, "Of course; there is no doubt about it."

Mr. Adley then seconded the adoption of the report on the Chairman's authority; he assured the meeting that he had absolute confidence in Mr. Cory-Wright and the committee, but he had not had time to read the report himself.

The Chairman: It was drawn up by twenty people.

Mr. Adley: I would rather take it on the authority of one or two; a report made by many people is generally absolutely unintelligible.

Further objection was taken to the mention, as if he were still living, of a member who had died. It would not, it was maintained, be done in the report of any railway company. The Chairman replied that this was not a railway company, but a hospital, and that the name in question was that of a member who had attended all the meetings during the year to which the report referred; he would not be a party to putting into a report for 1901 matters that happened in 1902. On Mr. Adley's putting the motion, the report was adopted.

The balance-sheet, which states that the bank account is overdrawn by £136 8s. 6d., was next criticised by Mr. J. P. Bevan, treasurer, who said that if the festival dinner raised £1,000 it would all be wanted to pay off the debt. He was understood to disapprove of the inclusion of £1,211 11s. 1d. (Diamond Jubilee Commemoration Fund and interest) as income; it made the funds look too prosperous. He wanted to see the hospital placed in a better position. The Chairman said he did not see that the hospital was going to the dogs because it was in debt on February 26th; it had paid its way, and he congratulated himself and the committee that it had done so.

There was no further discussion on the report, which states that a scheme is under consideration for bringing the charity under one control, thus avoiding the inconvenience of the dual management which has existed for the past three years. A special appeal is inserted, stating that, in view of the proposal of the L.C.C. to bring over 40,000 people from the congested districts of London, there ought to be at least 400 beds in the hospital.

The Medical Committee records its recognition of the marked improvement in the nursing department, due largely to the judicious, untiring devotion to duty of the lady superintendent, Miss E. Margaret Fox.

